# Avocado Peels and Seeds: Processing Strategies for the Development of Highly Antioxidant Bioplastic Films

**DOI:** 10.1021/acsami.1c09433

**Published:** 2021-08-04

**Authors:** Danila Merino, Laura Bertolacci, Uttam C. Paul, Roberto Simonutti, Athanassia Athanassiou

**Affiliations:** † Smart Materials, Istituto Italiano di Tecnologia, Via Morego, 30, Genoa 16163, Italy; ‡ Dipartimento di Scienza dei Materiali, Università di Milano-Bicocca, Via Roberto Cozzi 55, 20125 Milano, Italy

**Keywords:** nonedible waste, active food packaging, biodegradable, biomass hydrolysis, low methoxyl pectin

## Abstract

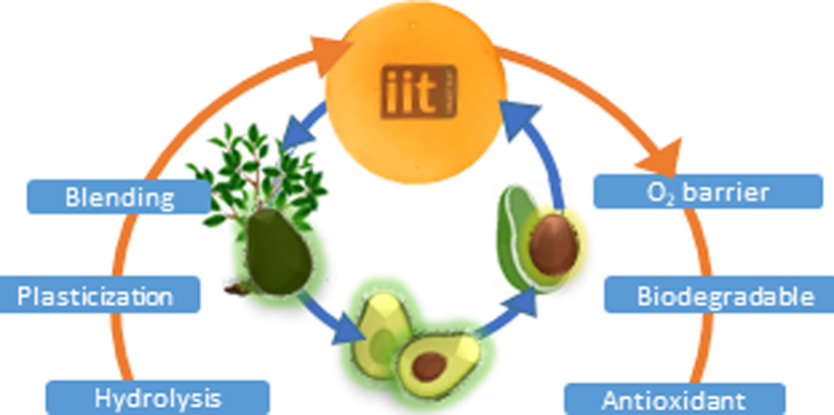

The industrial processing of avocados annually generates more than 1.2 million tons of avocado peels (APs) and avocado seeds (ASs) that have great potential in the production of active bioplastics, although they have never been considered for this aim until now. Separately, the APs and ASs, as well as a combination of avocado peels and seeds (APSs), were evaluated here for the first time for the preparation of antioxidant films, with application in food packaging. Films were prepared by casting, after their processing by three different methods: (1) hydrolysis in acid media, (2) hydrolysis followed by plasticization, and (3) hydrolysis and plasticization followed by blending with pectin polymers in different proportions (25 and 50 wt %). The results indicate that the combination of hydrolysis, plasticization, and pectin blending is essential to obtain materials with competitive mechanical properties, optical clarity, excellent oxygen barrier properties, high antioxidant activity, biodegradability, and migration of components in TENAX suitable for food contact applications. In addition, the materials prepared with APSs are advantageous from the point of view of the industrial waste valorization, since the entire avocado wastes are used for the production of bioplastics, avoiding further separation processes for their valorization.

## 1 Introduction

The recovery of biomass from inedible fruit and vegetable wastes for the preparation of bioplastics is a growing research trend due to its expected positive impact on the environment through the circular economy model.^1−4^ Bioplastics are defined as either biodegradable or biobased plastic materials or both. Nevertheless, nonbiodegradable bioplastics of biobased origin or biodegradable bioplastics from fossil sources principally follow a linear economy model and can hardly be considered sustainable. On the other hand, biodegradable bioplastics based on the use of biomass can offer the benefits of biodegradability and functional properties combined to a circular model in which waste is transformed into raw materials in the short term.^5,6^

The bioplastics obtained from the processing of plant residues, without carrying out extractive processes, are biocomposites composed of numerous natural polymers and phytochemicals that can provide new functionalities to the developed materials, such as antioxidant, antimicrobial, and nutraceutical properties.^7^ For this purpose, biomass is generally dried and milled to a powder form to inhibit the enzyme and bacterial activity and thus halt its decomposition during storage.^8^ Bioplastics cannot be directly obtained from this dried form, given the absence of thermoplasticity; therefore, different methods have been proposed for its dissolution or digestion.^2,4,9^ Among them, the acid hydrolysis stands out, which allows obtaining bioplastics through the disruption of plant cell walls, partial hydrolysis of constituent polymers, and release of cellulose microcrystals.^2^ This process commonly results in a viscous solution, and films are obtained by casting or other drying techniques. This method, especially when mild acids are used, is highly sustainable since the entire process is carried out in aqueous solutions, and the acid solution employed may be recovered by vapor collection during the drying of the films.

Avocado (*Persea americanaMill*) is a fruit that grows in tropical and subtropical climates and that year after year gains more acceptance, growing its consumption worldwide due to its numerous benefits for human health.^10,11^ Thus, world avocado production has been increasing for at least the last 40 years. In 2018, more than 6.4 million tons were harvested, and 918,531 ha of land were dedicated to its production in more than 60 countries.^12^ The avocado is produced throughout the year, and the Hass variety, the one used in this work, has been the most produced and marketed due to its quality, productivity, and constant availability.^13^

In addition to its direct consumption, this fruit is industrially processed for the production of oil, mashed avocado or guacamole, packaged slices and pieces, and dehydrated avocado, leaving the avocado peels (APs) and the avocado seeds (ASs) as residues, representing between 20 and 30 wt % of the fruit.^13,14^ These residues are rich in carbohydrates such as pectin, cellulose, hemicellulose, and starch and have a high potential for the production of value-added materials for the food, cosmetic, and pharmaceutical industries.^13^ Besides, they are an important source of bioactive compounds with high antioxidant activity, which makes them highly attractive for the production of active food packaging.^15^ Other uses reported in the literature include the use of ASs to produce biodiesel^16,17^ or for the production of carbonized materials used as absorbents.^18^ However, there is no information in the literature on the application of avocado byproducts in the packaging field. Most of the publications that seek to value the industrial avocado waste (AW) are based on the use of the seeds, leaving aside the value that the peels can provide.^19,20^ In this work, strategies have been developed to valorize both byproducts of avocado processing, to prepare bioplastics with high antioxidant activity.

In particular, a method for processing AW to obtain functional films, studying the properties of the avocado’s residues individually (APs and ASs) and in combination (avocado peels and seeds, APSs), has been established. For that, three different processing strategies were investigated: (1) acid hydrolysis, (2) acid hydrolysis followed by plasticization, and (3) acid hydrolysis, followed by plasticization and subsequent blending with pectin, a polysaccharide naturally present in plant cell walls and commonly used as a binder agent.^1^ Pectin is an interesting polysaccharide, given its structural characteristics, renewability, and biocompatibility, commonly extracted by treating citrus peels with acidified water, highlighting that its production does not compete with the lands destined for food production, as is the case for other bioplastics.^22^

Using the abovementioned methods, the authors have developed different biocomposite films with various properties. The obtained films were analyzed for their mechanical, water vapor/oxygen barrier, optical, and morphological properties, as well as their biodegradability and suitability as antioxidant food packaging.

## 2 Materials and Methods

### 2.1 Materials

Avocado seeds and peels were obtained from ripe avocado fruits of the Hass variety purchased at a local grocery store and imported from Chile (Subsole Avocados S. A). AW was obtained after the edible part was removed with a spoon. The APs and ASs were hand-washed with tap water and stored at −18 °C. After that, they were dried in an oven at 40 °C for 48 h and finally ground to powder using a blender (Oster, VERSA). The grounded avocado peels and seeds were finally passed through a sieve of 300 μm size (VWR International) in order to obtain a fine powder with a particle size of less than 300 μm.

The polyglycerine-3 (G3) used as a plasticizer was obtained from Spiga Nord S. p. A. (Italy). It is composed of 15–30% diglycerol, 35–48% triglycerol, 10–25% tetraglycerol (polymers of glycerol repeating units with polymerization degrees of 2, 3, and 4), and less than 10% of other components including glycerol and heptaglycerol. Low methoxyl pectin (LMP) from citrus peel with 83 wt % galacturonic acid units and 7.7 wt % methoxyl groups, acetic acid, CaCl_2_, ethanol 96%, and the 1,1-diphenyl-2-picrylhydrazyl (DPPH) radical used in the antioxidant assay were purchased at Sigma-Aldrich and used without further purification.

### 2.2 AW-Processing Strategies

AP and AS powders obtained using the procedure described in Section 2.1 were used for the preparation of AP- and AS-based films. APS powder used for the preparation of APS-based films was prepared by the combination of 35 wt % APs and 65 wt % ASs, which is their average proportion in the dried avocado fruit waste.

#### 2.2.1 Acid Hydrolysis

AP, AS, or APS powder (5 wt %) was added in 1 M aqueous solution of acetic acid and stirred for 24 h at 30 °C. After that, the obtained solutions were poured into Petri dishes previously covered with polydimethylsiloxane (PDMS) to facilitate the peel-off process after their drying under a fume hood at room temperature for 48 h. Alternatively, AP, AS, or APS solutions stirred for 24 h at 30 °C in 1 M acetic acid were subsequently heated at 80 °C and kept under stirring for 1 h. The latter process was selected to improve the starch granules structure’s disruption since avocado seed starch gelatinization temperature was reported to be in the range of 76^23^–78 °C.^20^ The obtained solutions were again poured into Petri dishes to obtain films after drying for 48 under a fume hood at room temperature.

#### 2.2.2 Acid Hydrolysis Followed by Plasticization

Once completed, the 24 h of hydrolysis at 30 °C as described in Section 2.2.1, 30 wt % G3 plasticizer with respect to the AP, AS, or APS powder was added to the solutions, the temperature was increased to 80 °C, and the mixture was kept under stirring for 1 h. After that, the films were prepared by casting as previously described (Section 2.2.1). The obtained samples based on APs, ASs, or APSs were named as follows: AP-30G3, AS-30G3, and APS-30G3, respectively.

#### 2.2.3 Blend Preparation

AP, AS, and APS solutions after hydrolysis and G3 plasticizer addition were blended with different amounts (25 and 50 wt % with respect to the AW/G3) of LMP. For that, solutions prepared in Section 2.2.2 were combined with an appropriate amount of a 2 wt % solution of LMP in deionized water and with a 2 wt % CaCl_2_ aqueous solution to obtain pectin cross-linking, using the stoichiometric ratio *R* = 2[Ca^2+^]/[COO^–^] = 1. After stirring, the solutions were poured into Petri dishes covered with PDMS, and they were allowed to dry under the hood for 48 h. The obtained dried films were named as indicated in Table 1.

**Table 1:** Sample Names and Composition^a^

name	AP (wt %)	AS (wt %)	APS (wt %)	G3 (wt %)	Ca-LMP (wt %)
AP-30G3-25LMP	45			30	25
AP-30G3-50LMP	20			30	50
AS-30G3-25LMP		45		30	25
AS-30G3-50LMP		20		30	50
APS-30G3-25LMP			45	30	25
APS-30G3-50LMP			20	30	50

a Abbreviations: AP: avocado peel; AS: avocado seed; APS: avocado peel and seed; G3: polyglicerine-3; and LMP: low methoxyl pectin.

### 2.3 Characterization of AW and Avocado Films

#### 2.3.1 Solid-State NMR

The ^13^C solid-state NMR spectra of the different AWs were obtained with an Avance 400 spectrometer (Bruker) at 100 MHz using magic angle spinning (MAS) with a 4 mm rotor and a spinning rate of 10 kHz.^24^ The cross-polarization (CP) ^13^C MAS NMR spectra exploited the transfer of polarization under Hartmann-Hahn conditions obtained by ramped amplitude variation of the spin locking field (RAMP)^25^ and TPPM decoupling.^26^ A contact time of 2.5 ms and a recycling delay of 2 s were used, and 8192 scans were acquired for all the samples.

Since the NMR technique is not able to differentiate between hemicellulose and starch, the starch present in the AW was removed following the method reported by Chel-Guerrero *et al.*(27) Briefly, a 20 wt % solution of AS or AP powder was suspended into a sodium bisulfite solution (2.4 g/L), and the suspension was stirred for 1 h. After that, the starch granules, small water-soluble molecules, and small powder particles were separated from the fibers by filtering through a 50-mesh screen. The AP and AS fibers were collected, dried in an oven for 24 h at 50 °C, and analyzed again by NMR. Thus, the amount of hemicellulose was assessed and so, indirectly, the amount of starch.

#### 2.3.2 Films’ Thickness Measurements

The thickness of all film samples was measured in 10 different random points with a digital micrometer (Mitutoyo, United States of America) with 0.001 mm sensitivity. The average film thickness was used for the calculations in the experiments of mechanical, optical, and barrier properties.

#### 2.3.3 Films’ General Appearance and Optical Properties

Films were visually analyzed, and photographs were taken in order to show their general appearance. The films’ optical properties were analyzed by measuring the light transmittance in the 200–800 nm range using a Cary 6000i UV–vis–NIR spectrophotometer (Varian Inc). After that, all the spectra were normalized to a film thickness of 100 μm. At least two spectra of each sample were measured, observing good reproducibility.

#### 2.3.4 Scanning Electron Microscopy

AP-, AS-, and APS-based films were analyzed in a JEOL JSM-6490LA microscope, working at an acceleration voltage of 10 kV and a filament current of 78 μA. For the cross-sectional analysis, films were cryofractured by immersion in liquid nitrogen, and samples were fixed to aluminum stubs with carbon tape. Before the analysis, samples were coated with 10 nm of gold to assure their conductivity.

#### 2.3.5 Fourier Transform Infrared Spectroscopy

Fourier transform infrared spectroscopy (FTIR) spectra of powders and films were collected in a VERTEX 70v Bruker spectrophotometer using the attenuated total reflectance (ATR) with a diamond crystal accessory. For that, spectra were acquired in the 4000–600 nm range at 4 cm^–1^ spectral resolution and 64 scans.

#### 2.3.6 X-ray Diffraction

X-ray diffractograms were measured using a PANalytical Empyrean X-ray diffractometer with CuKα radiation (λ = 1.54178 Å) at a scanning rate of 0.08°/s, ranging from 5 to 60°. The operation voltage and current were maintained at 40 kV and 35 mA, respectively.

#### 2.3.7 Mechanical Properties

Mechanical characterization was performed using uniaxial tensile tests in an INSTRON 3365 universal testing machine. The crosshead initial distance was fixed to 35 mm, and the speed was 3 mm/min. Film samples were cut to a dog-bone shape with the straight part measuring 25.01 mm in length and 3.98 mm width. Before the tests, the specimens were conditioned overnight in an Espec SH-262 environmental chamber at 50% relative humidity (RH) and 24 °C. The values of Young’s elastic modulus (*E*), tensile strength (σ), and elongation at break (ε_b_) were obtained from the stress (MPa) *versus* strain (mm/mm) curves, and results are reported as average ± SD.

#### 2.3.8 TGA

The materials developed were studied in a TA Q500 thermogravimetric analyzer under a nitrogen atmosphere at a 50 mL/min flow rate. Samples (approx. 10 mg) were heated at a rate of 10 °C/min from room temperature to 900 °C, and their thermogravimetric (TGA) and first-derivative TGA (DTGA) curves were analyzed.

#### 2.3.9 Water Contact Angle

Static contact angle measurements were performed in a Theta Optical Tensiometer (Dataphysics OCAH200) using 3 μL droplets of ultrapure Milli-Q water. At least 10 contact angles were measured for each film, and they were reported as average ± SD.

#### 2.3.10 Water Vapor Permeability

For water vapor permeability (WVP) determination, aluminum-based permeation capsules with an internal diameter of 6 mm containing 400 μL of Milli-Q water (100% RH) were covered with the samples and sealed with two O-rings and a ring-shaped cap adjusted with screws. Test capsules were stored in a chamber with dried silica gel in order to simulate 0% RH conditions. Changes in the capsule weight were monitored for 5 h. The slope obtained from the capsule weight loss (g) *versus* time (s) plot was divided by the exposed film area (m^2^) for the determination of the water vapor transmission rate (WVTR), and it was used in eq 1 for the determination of the WVP (g/m s Pa).
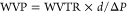
1where *d* is the average thickness of each sample (m) and Δ*P* (Pa) is the difference in vapor pressure through the film. All samples were analyzed in triplicate, and results were expressed as average ± SD.

#### 2.3.11 Oxygen Permeability

The films’ oxygen permeation analysis was performed using an Oxysense 5250i device (Massachusetts, USA) equipped with a film permeation chamber. This machine was operated according to ASTM test Method F 3136-15. The test was performed under standard laboratory conditions, that is, 21 ± 2 °C and 50 ± 2% RH. The permeation chamber consisted of a cylinder divided into two parts (sensing well and driving well). At first, the sample was placed over the sensing well, and the chamber was correctly closed using the locking bolts. The sensing well was instrumented with a fluorescence sensor (oxydot) mounted on the chamber’s nitrogen purged side, while the driving well was kept open to ambient air. The oxygen gas transmission rate (O_2_TR) of the film was measured using the oxysense permeability (O_2_P) analyzer with a fluorescence sensor. When oxygen passes from the driving well through the films to the sensing well, the fluorescence is quenched, decreasing its lifetime proportionally to the oxygen concentration. Oxysense software measures this oxygen concentration over time (O_2_TR). At least 10 recorded values were taken for each sample with a minimum coefficient of determination value (*R*^2^) of 0.995. The oxygen permeability (O_2_P) of the film was then calculated according to eq 2
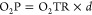
2where O_2_P (cm^3^ μm m^–2^ day^–1^ kPa^–1^) is the oxygen permeability, O_2_TR (cm^3^ m^–2^ day^–1^ kPa^–1^) is the oxygen transmission rate, and *d* (μm) is the average thickness of the sample.

#### 2.3.12 Biodegradability in Seawater

The biodegradability of the samples was assessed by means of biochemical oxygen demand (BOD), which can be easily determined by monitoring the oxygen consumption in a closed respirometer. In detail, about 25 mg of each sample was added to 432 mL of seawater as the single carbon source. The seawater was chosen in order to mimic real environmental conditions. It already contains microbial consortia and the saline nutrients needed for their growth.

The experiment was conducted at room temperature inside dark glass bottles with a volume of 510 mL, hermetically closed with the OxiTop measuring head. A CO_2_ scavenger was added in order to sequestrate carbon dioxide produced during the biodegradation, and biotic consumption of the oxygen present in the free volume of the system was measured as a function of the decrease in pressure.

According to ISO 14851i, raw data of oxygen consumption (mg O_2_/L) were corrected by subtracting the blank’s value, obtained by measuring the oxygen consumption of the seawater in the absence of any test material. After this subtraction, values were normalized on the individual sample’s mass and referred to 100 mg of the material (mg O_2_/100 mg material) and plotted *versus* time.

### 2.3.13 Antioxidant Capacity

The antioxidant capacity of selected films was evaluated against the DPPH^•^ radical. DPPH^•^ radical scavenging assay was carried out following the methodology reported by Guzman-Puyol *et al.*(28) Discs of 16 mm diameter (approximately 0.05 g) of the selected samples were added to 4 mL of 0.05 mM DPPH solution in ethanol. The decrease in the absorbance of the solution due to the antioxidant films’ action was monitored at 517 nm during 24 h in a Cary 6000i spectrophotometer. The obtained absorbance values were used to calculate the radical scavenging activity (RSA), as indicated in eq 3.
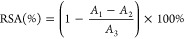
3where *A*_1_ is the absorbance of the film-containing solutions at 517 nm and at different times and *A*_2_ is the measured absorbance of the film-containing solutions in ethanol at 517 nm. *A*_3_ is the measured absorbance of the control at 517 nm (2 mL of DPPH solution in ethanol).

All measurements were performed in duplicate, and results were expressed as average ± SD. AP and AS powders were also analyzed, and curves were normalized according to their amount present in the film samples.

With the aim of comparing the antioxidant results found in this work with others in the literature, results were also expressed as Trolox (6-hydroxy-2,5,7,8-tetramethylchroman-2-carboxylic acid) equivalent antioxidant capacity (TEAC), expressed as μmol of Trolox/g of the dried film.

#### 2.3.14 Overall Migration of Components in Tenax

The migration of components from the different films into the food was tested using Tenax as a simulant for dry food. Round samples of 20 mm diameter were put in clean glass vials with 80 mg of Tenax and stored in the oven for 2 h at 70 °C. The overall migration was obtained by calculating the mass difference of Tenax before and after the treatment.

### 2.3.15 Statistical Analysis

Results were reported as mean ± SD. The one-way analysis of variance (ANOVA) and Tukey’s test were applied to determine the significance in differences among the mean values at a 0.05 level of significance using the Origin 2019b program.

## 3 Results and Discussion

### 3.1 AW Chemical Composition Assessed by Solid-State NMR

The approximate polymer composition of AW was assessed by solid-state NMR, and the results are depicted in Table 2. NMR curves and interpretation of the displacements are included as Supporting Information, Figure S1. Results obtained indicate that the peel has a higher content of fiber than the seed, in agreement with the results reported by other authors.^29^ The amount of starch detected here for ASs was a bit inferior to the 30 wt % reported by Araujo *et al.*(13)

**Table 2:** Approximate Composition of the APs and ASs Belonging to the Hass Variety Determined by Solid-State NMR as Described in Section 2.3.1

material^a^	cellulose (mol %)	hemicellulose (mol %)	lignin (mol %)	pectin (mol %)	starch (mol %)	polyesters (mol %)
AP	38	37	10	14		1
AS	21	37	5	13	23	1

a Abbreviations: AP: avocado peel and AS: avocado seed.

Other authors have reported that the water- and ethanol-extracted contents of avocado are close to 35 wt % and that they contain a lot of active compounds, including flavonoids, phenolics, steroids, tannins, and terpenoids, among others.^30^ For a detailed chemical composition of the extractives and active compounds, the reviews of Araújo *et al.*(13) and Jimenez *et al.*(14) are highly recommended.

### 3.2 Acid Hydrolysis for AW-Based Film Preparation

**Figure 1:**
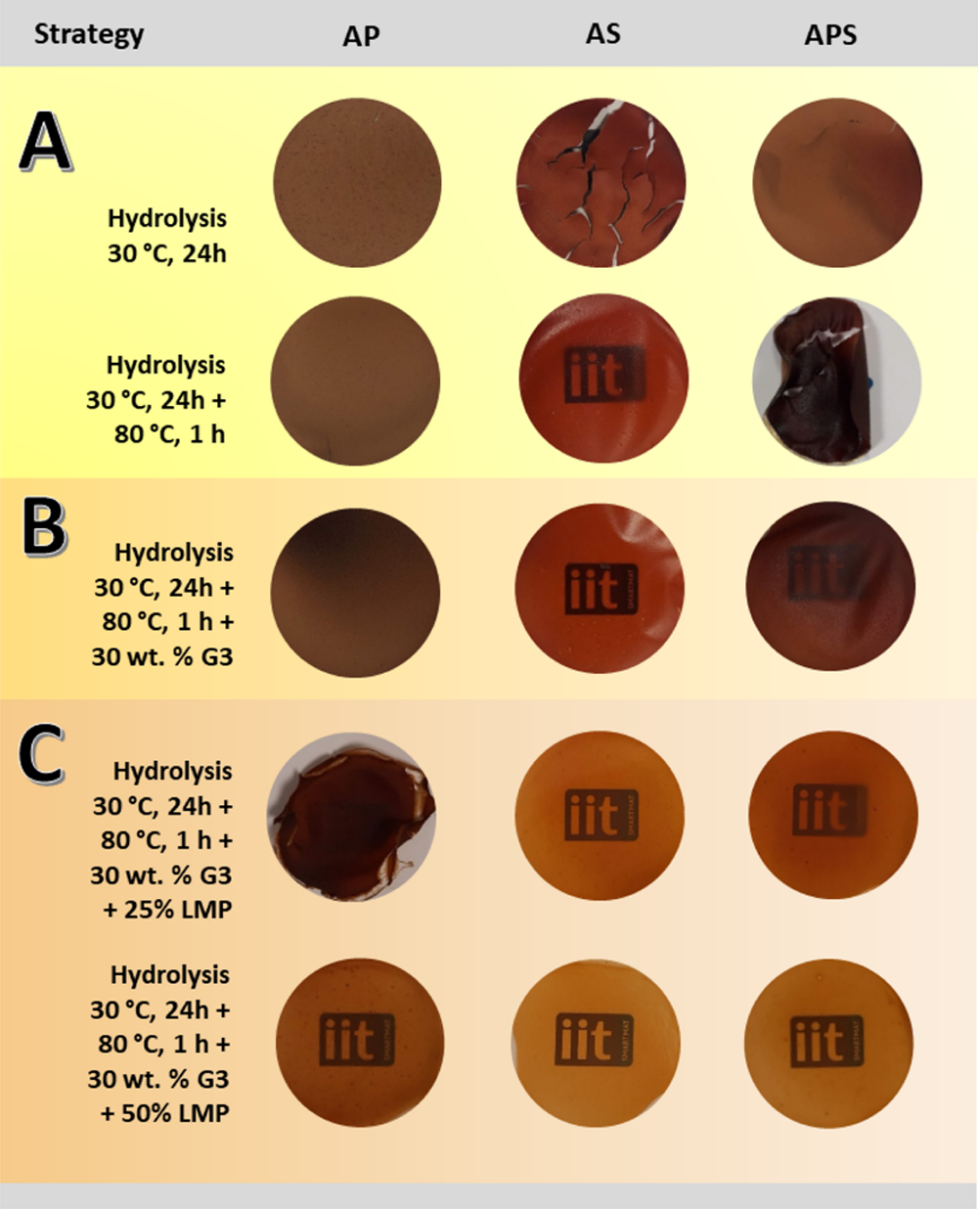
Photographs of the developed films obtained after following three different processing strategies. (A) Acid hydrolysis, (B) acid hydrolysis followed by plasticization, and (C) preparation of composites with cross-linked LMP. The authors’ institute logo was placed under the developed films to show their optical clarity.

Photographs of the films obtained after AP, AS, and APS hydrolysis are included in Figure 1A. The acid hydrolysis strategy at 30 °C of AW components has led to very brittle films with a clearly poor cohesion between particles. These materials were not further analyzed. Instead, different steps were pursued in order to improve their properties. The first one consisted on the subsequent heating of the hydrolysates at a higher temperature of 80 °C for 1 h to favor further hydrolysis and gelatinization of the starch present in ASs and APSs.^20,23^ The obtained films presented improved aspect, but they were still very brittle and were not further analyzed. The next step entailed the addition of the G3 plasticizer, which has been previously used for the plasticization of potato peel, demonstrating that it can lead to materials with superior mechanical properties compared to the ones obtained with the traditionally used glycerol.^3^

### 3.3 Hydrolysis Followed by Plasticization for AW-Based Film Preparation

Hydrolyzed solutions for 24 h at 30 °C and subsequently for 1 h at 80 °C, as also described in Section 3.2, were mixed with 30 wt % G3, with respect to the AP, AS, or APS powder, in order to improve the developed films’ properties. The visual appearance of the films, after the solvent evaporation, improved significantly with respect to the nonplasticized films, as can be seen in Figure 1B.

**Figure 2:**
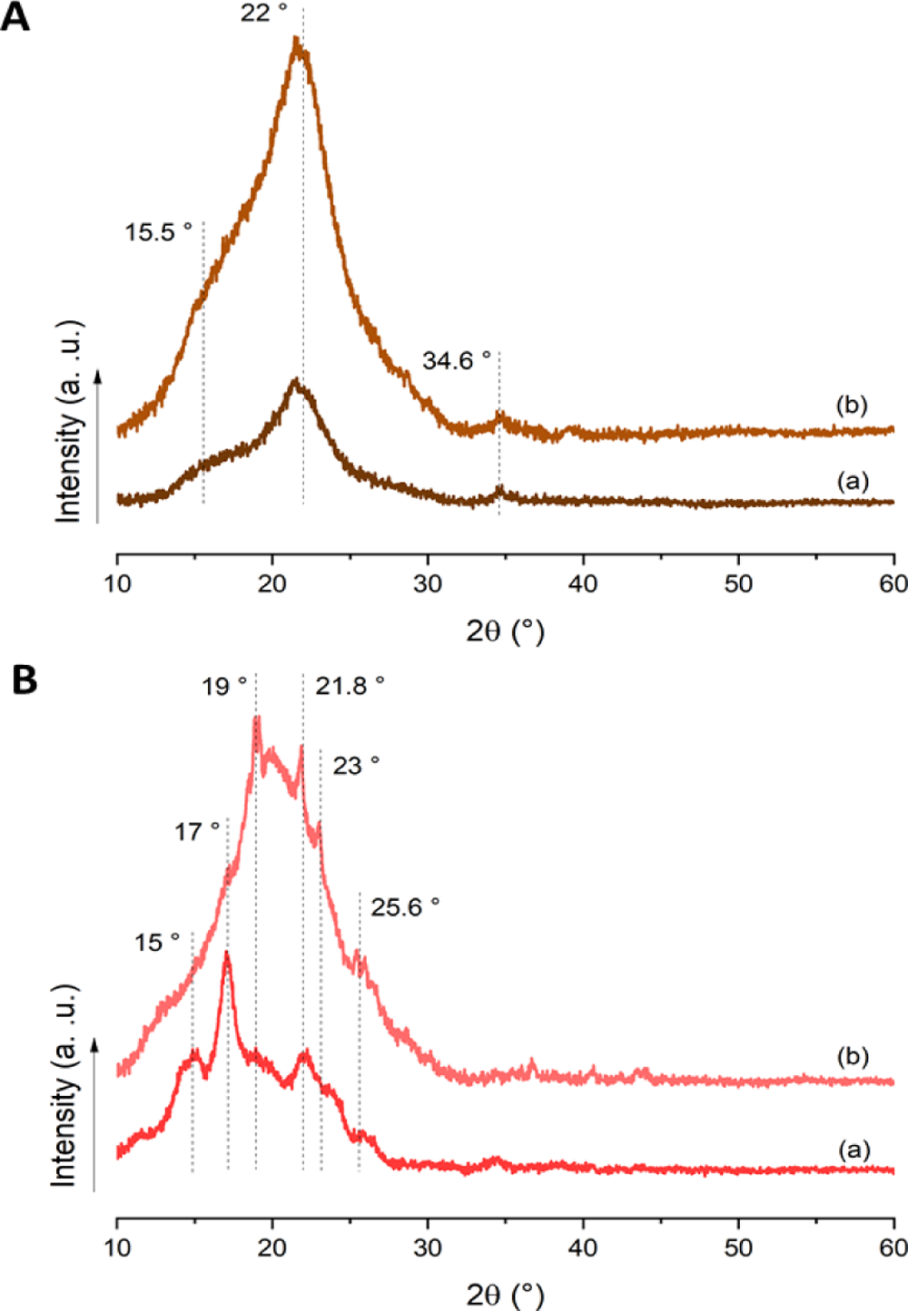
XRD patterns of (A) AP powder (a) and AP-30G3 film (b) and (B) AS powder (a) and AS-30G3 film (b).

The increase in the amorphous part, at the expense of crystallinity, of the plasticized films is demonstrated in Figure 2. In particular, Figure 2A(a) shows that AP powder presented the typical X-ray diffraction pattern obtained for cellulose I polymorph (2θ = 15 and 22.6°).^31^ Peaks with a maximum at 15.5, 22.0, and 34.6° were identified and associated with the (1 1 0), (2 0 0), and (0 4 0) lattice planes, respectively, of native cellulose.^22^ As explained by Barnette *et al.*, amorphous hemicellulose and lignin do not display any diffraction peaks but a diffuse scattering halo centered at 18°, along with the 12–27° 2θ range, overlapping cellulose crystalline peaks.^32^ Similar X-ray diffraction patterns were reported for switchgrass,^33^ raw wood chip samples of scarlet oak,^32^ a hybrid grass variety from India,^34^ and sugar palm fibers.^31^ The AP powder hydrolysis followed by plasticization effectively diminishes the crystallinity in the AP-based films, as shown from the increase in the amorphous halo in Figure 2A(b), although the distinct peaks of cellulose can be still visible.

On the other hand, the X-ray diffraction pattern of AS powder, shown in Figure 2B(a), presented the typical B-type crystalline pattern of starch with peaks at 15.0, 17.0, 19.0, 21.8, 23.0, and 25.6° (2θ), in agreement with the results of dos Santos *et al.*,^35^ Macena *et al.*,^36^ and Kahn.^37^

The reduction in the peak intensities after hydrolysis and addition of G3 can be observed in Figure 2B(b), mainly on the peaks appearing at 15° and 17°, while a significant increase in the amorphous halo was observed, suggesting the effective plasticization of avocado starch after the followed methodology.^38^ Finally, in Figure S2 included in Supporting Information, it can be seen that the APS-30G3 sample presented a very low crystallinity as a result of the combination of both AS and AP hydrolyzed and plasticized biomass.

The infrared spectra of the AP and AS powders and of the plasticized films derived from them are presented in Figure S3 included in Supporting Information. The bands observed between 3700 and 3000 cm^–1^ were associated with the stretching of the O–H groups present in polysaccharides, the main components of AW,^29^ and in carboxylic acids, alcohols, phenols, and adsorbed water molecules.^39^ The region beyond 3000 cm^–1^ and up to 2800 cm^–1^ shows the doublet of the C–H stretching bands of CH_2_ groups corresponding to the symmetric and asymmetric stretching modes usually associated with the presence of carbohydrates and lipids.^22,39^ The bands between 1800 and 1500 cm^–1^ are due to several overlapping modes of vibration, among which the following can be identified: the presence of carbonyls from fatty acid ester groups (1740 cm^–1^), the bending of adsorbed water molecules (1640 cm^–1^),^40^ the amide I (1700–1600 cm^–1^) and amide II (1565–1520 cm^–1^) bands present in proteins,^39^ and the bands associated with the angular deformations of the −CO– and −C=C– groups present in lignin or other phenolic compounds (1596–1515 cm^–1^).^22^ Finally, in the fingerprint area (1200–800 cm^–1^) appeared the characteristic bands of symmetric stretching of the C–C, C–OH, and C–O–C groups typical of carbohydrates.^22,39^

After the addition of G3, the peaks assigned to the stretching of the groups mentioned above changed their position and relative intensity. The observed shifts were attributed to new interactions by hydrogen bonds between the hydrolyzed polysaccharides present in APs and ASs and the plasticizer, G3. New peaks’ appearance was not evidenced.^41^

**Figure 3:**
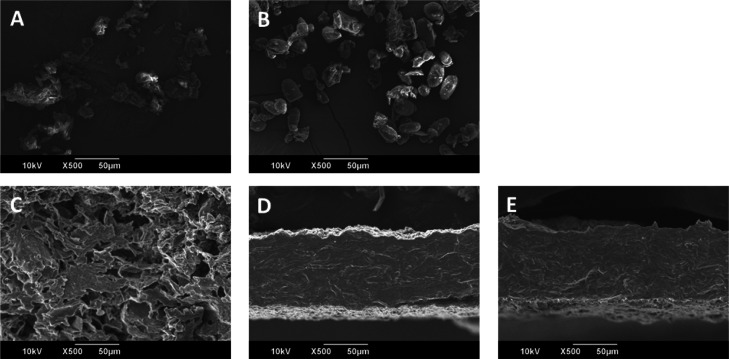
SEM micrographs of (A) AP powder, (B) AS powder, and the cross-sectional surface of (C) AP-30G3, (D) AS-30G3, and (E) APS-30G3. The scale bar represents 50 μm, and images were taken with ×500 magnification.

SEM images of AP and AS powder are included in Figure 3A,B, respectively. AP powder presented particles of irregular shape, while for AS powder, a smooth oval shape was identified and associated with avocado starch, together with the presence of other compounds of irregular nature.^23^ According to Bet *et al.*,^23^ avocado starch granules have a diameter of 21 μm, similar to the 5–35 μm reported by Kahn^37^ and in accordance with the 25.1 ± 4.2 μm observed in this work.

Figure 3C shows that the cross-sectional area of AP-30G3 presented a porous and foamy structure, suggesting low cohesion among material components, also observed macroscopically. In contrast, AS-30G3 (Figure 3D) and APS-30G3 (Figure 3E) presented a relatively homogeneous and compact cross section, suggesting good interaction among components and excellent compatibility between processed ASs and APs.

Besides, the difference in the various films’ thickness can be noticed from the micrographs, although the amount of material was constant in all cases. In particular, while nonsignificant differences were observed among the thickness of AS-30G3 and APS-30G3 (123 ± 15 μm and 150 ± 34 μm, respectively), the AP-30G3 samples presented a significantly superior thickness (253 ± 39 μm), indicating the lack of cohesion among the various components, the presence of voids in the films, and the necessity of a binding agent.^42^

The mechanical properties, water contact angle (WCA), and WVP of the plasticized films are demonstrated in Table 3. The film AS-30G3 presented the best mechanical properties, likely due to the gelatinization of the starch and the formation of a self-assembled composite with homogenously distributed cell wall components present in ASs. On the other hand, the films obtained from AP’s plasticization presented inferior mechanical properties associated with their high fiber content and porous structure, as evidenced by NMR and SEM, respectively.

**Table 3:** Mechanical Properties, WCA, WVP, and Antioxidant Capacity of AW and AW-LMP Films^a^^,^^b^

	Young’s modulus (MPa)	tensile strength (MPa)	elongation at break (%)	WCA (deg)	WVP (× 10^–9^ g/s m Pa)	DPPH scavenging activity (μmol TE/g dried sample)
AP powder						26.4 ± 0.2^c^
AS powder						25.9 ± 0.7^c^
AP-30G3	38.6 ± 4.8a	0.6 ± 0.04a	3.9 ± 1.6a	0^a^	2.6 ± 0.1^c^	not assessed
AS-30G3	113.6 ± 4.7^b^	3.4 ± 0.07^c^	19.8 ± 1.3^b^	0^a^	1.1 ± 0.2^a^	not assessed
APS-30G3	59.9 ± 20.2^a^	1.8 ± 0.2^b^	12.2 ± 0.5^b^	0^a^	1.4 ± 0.2^a,b^	not assessed
AP-30G3-25LMP	229.5 ± 41.6^c^	9.1 ± 0.4^b^	16.9 ± 3.4^b^	100.9 ± 6.7^d^	2.7 ± 0.2^c^	not assessed
AS-30G3-25LMP	252.6 ± 15.1^c,d^	10.7 ± 1.4^b^	17.1 ± 3.6^b^	59.8 ± 5.0^c^	1.6 ± 0.01^a,b^	not assessed
APS-30G3-25LMP	256.8 ± 26.0^c,d^	10.4 ± 0.9^b^	17.9 ± 3.1^b^	106.3 ± 5.1^d,e^	1.7 ± 0.3^a,b^	not assessed
AP-30G3-50LMP	313.7 ± 28.6^d,e^	16.9 ± 2.1^c^	19.7 ± 3.9^b^	108.3 ± 4.7^e^	2.5 ± 0.4^c^	4.7 ± 0.5b
AS-30G3-50LMP	460.1 ± 8.1^f^	18.5 ± 1.3^c^	13.8 ± 2.4^b^	48.4 ± 7.3^b^	1.2 ± 0.09^a,b^	3.3 ± 0.2a
APS-30G3-50LMP	341.7 ± 46.3^e^	17.9 ± 2.7^c^	16.8 ± 6.1^b^	106.1 ± 3.7^d,e^	1.8 ± 0.06^b^	3.4 ± 0.2a

a Equal letters indicate that the results are not significantly different according to Tukey’s test (*p* < 0.05).

b Abbreviations: AP: avocado peel; AS: avocado seed; APS: avocado peel and seed; G3: polyglicerine-3; LMP: low methoxyl pectin; WCA: water contact angle; WVP: water vapor permeability; DPPH: 1,1-diphenyl-2-picrylhydrazyl radical; and TE: Trolox equivalent.

The films obtained from the combination of ASs with APs, that is, APS-30G3, presented an intermediate behavior. Although the addition of APs, rich in plant fibers, to ASs, a starchy matrix, is expected to increase the mechanical resistance of the developed materials, this effect is usually attained when the fibers are preprocessed.^43^ In particular, natural fibers are normally covered by fats, waxes, lignin, pectin, and hemicelluloses, so typically, a bleaching or alkaline treatment is performed to remove these external components, depolymerize the amorphous cellulose, and release the short-length crystals, which in turn improve mechanical, barrier, and thermal properties of the composites.^44^ Nevertheless, the AP and AS combination was found to improve the properties of AP-30G3 significantly, showing that ASs positively contributed to the compatibilization and binding of the components present in AP-30G3, as supported by SEM micrographs.

Regarding the bioplastic interaction with water vapor, APS-30G3 and AS-30G3 presented the lowest values of WVP. These results can be due to the compact and homogeneous structure that they presented, as shown in Figure 3D,E, and the good compatibility of components, evidenced by the formation of hydrogen bonds, as revealed by FTIR (Figure S3). AP-30G3 films, in contrast, presented a porous structure that could facilitate the water vapor molecules’ penetration into the matrix, increasing their WVP. With regard to the WCA, all the materials were highly hygroscopic since the water drops were totally absorbed by the films.

### 3.4 Addition of LMP for AW-Based Composite Film Preparation

Depending on their composition, films composed exclusively of vegetable residues may have low mechanical and barrier properties and, sometimes, even lack consistency. For this reason, their combination with binding agents has been used for the development of higher-performance composites.^1,42^ Pectin has been proved a suitable biopolymer for this purpose in edible fruit and vegetable films.^1^ The films prepared from this polymer have shown good oxygen barrier and mechanical properties when plasticized and are low cost, highly available, and biodegradable.^45^ Therefore, to improve AW-based films’ performance, compounds were prepared with pectin in two different concentrations: 25 and 50 wt % with respect to the AW-G3 content.

As shown in Figure 1, nicely compact films were developed for all the LMP/AS-G3 and LMP/APS-G3 ratios investigated and for the AP-G3 with 50 wt % LMP. The casting method has proven to be an effective procedure to obtain films with homogeneous and smooth surfaces, with the absence of defects such as pores or cracks. In the same figure, it can be seen from the clarity of the illustration of the logo of the authors’ institute placed under the samples that the optical transmittance of the films increased in all cases with the increase in the amount of cross-linked LMP in the formulation. These results were in line with the optical transmittance spectra of the films, evaluated by UV–vis spectroscopy in the 200–800 nm range and included in Supporting Information, Figure S4.^40^

The optical differences observed among different AW components (ASs and APs) are related to their chemical composition. AS-30G3-25LMP and AS-30G3-50LMP films presented the highest optical clarity, followed by the APS-30G3-25LMP and APS-30G3-50LMP films, while the AP-30G3-25LMP and AP-30G3-50LMP films had the poorest optical clarity. Most likely, in the AP films, the increased presence of scattering centers, such as microfibers and microvoids, is responsible for the increase in these materials’ opacity.^46^

UV–vis absorption spectra of AP-30G3 and AS-30G3 display 0% transmittance or 100% absorption in the 200–360 nm range, which is due to the presence of cyclic organic molecules typically absorbing in that region, such as coumarins, saponins, alkaloids, tannins, reducing sugars, catechins, epicatechins, flavonoids, and polyphenolic compounds,^47^ whose main functional groups were identified by FTIR.

Cross-linked LMP presented similar FTIR bands to the AW powders and films (Figure S3). O–H groups’ stretching vibration at 3339 cm^–1^ and C–H groups’ stretching in CH_3_ and CH_2_ overlapped in the 3000–2800 cm^–1^ range. LMP also presented bands at 1737 and 1603 cm^–1^ associated with the C=O stretching in methoxyl ester groups (−COOCH_3_) and the carboxylate ion’s asymmetric vibration (COO^–^) stretching, respectively.^48,49^ As can be seen, the carboxylate ion band’s intensity is higher than that of carboxyl groups, which is characteristic of LMP.^41^ The peaks that appear centered at 1143, 1096, and 1007 cm^–1^ in the fingerprint area are typically assigned to the coupled stretching vibrations of C–O–C, C–C, and C–OH of the polygalacturonic acid backbone of pectin.^41,50^

After the addition of LMP, many changes were observed in the FTIR spectra of the AP, AS, and APS composite films in terms of peak positions and intensities (Figure S3). The O–H and C–H stretching peaks suffered from slight shifts, and the peak assigned to carbonyl stretching vibration increased as LMP content increased in the composites. In addition, changes in the fingerprint region were observed for the composites, which started to present the typical polygalacturonic backbone bands associated with pectin.

All the shifts observed in the spectra may be attributed to the interactions between pectin and AW components *via* the formation of hydrogen bonds since both components were based on polysaccharide chemical structures.^41,51^ Similar shifts were observed for films prepared by the combination of pectin and thermoplastic starch.^51^

**Figure 4:**
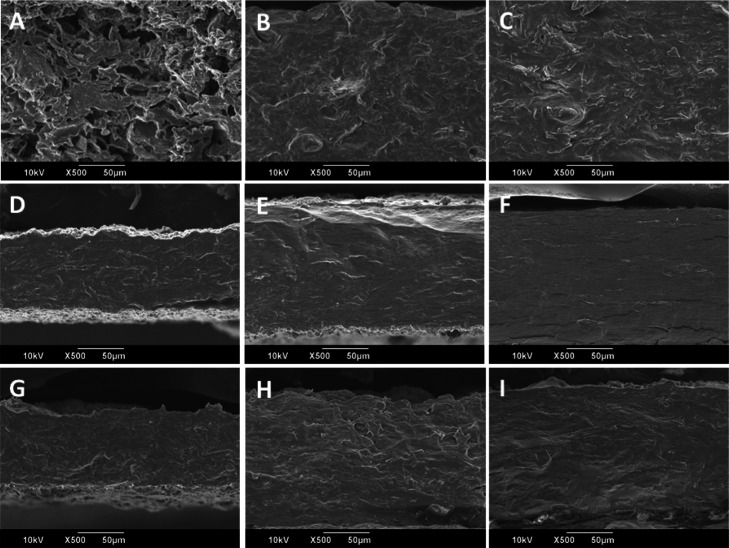
SEM micrographs of (A) AP-30G3, (B) AP-30G3-25LMP, (C) AP-30G3-50LMP, (D) AS-30G3, (E) AS-30G3-25LMP, (F) AS-30G3-50LMP, (G) APS-30G3, (H) APS-30G3-25LMP, and (I) APS-30G3-50LMP.

The cross-sectional surface of the composites was observed by SEM, and the micrographs are shown in Figure 4. The addition of LMP led to a homogeneous fracture surface with a uniform distribution of components and the absence of agglomerates. Similar results were reported for starch films with pectin particles and cotton fibers.^51^ The most significant changes were observed in the case of APs, which considerably improved the distribution of its components, demonstrating, as with FTIR, a good affinity with LMP. In general, no significant changes were observed in the appearance of the fracture surfaces with the increase in the LMP content. However, a slight increase in the films’ thickness was identified in the AS and APS films and associated with the proposed “egg-box” structure of LMP and its conformational changes due to AW’s interaction. Similar observations were reported for pectin films with malt bagasse fibers.^22^

**Figure 5:**
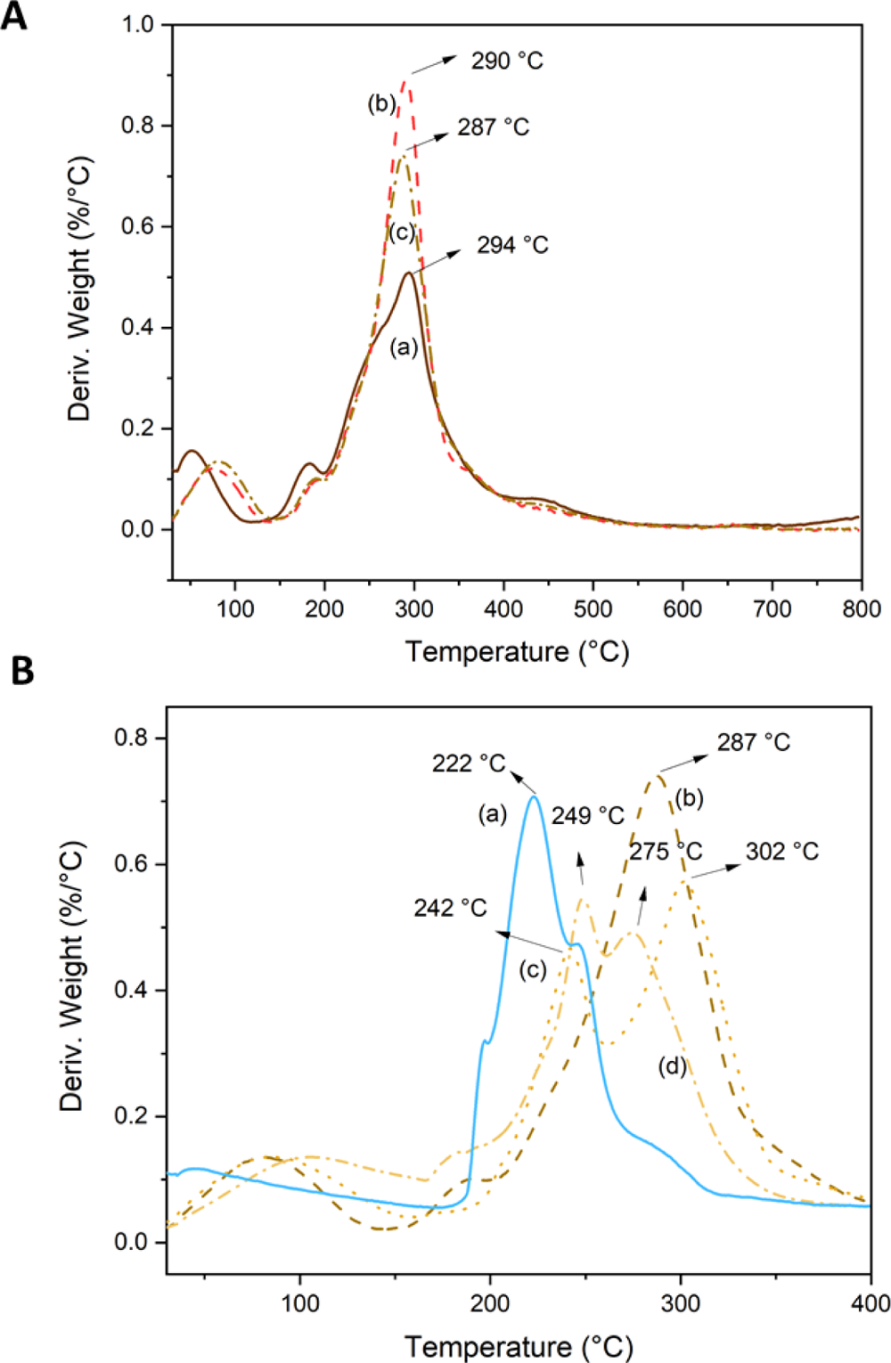
DTGA curves of (A) AP-30G3 (a), AS-30G3 (b), and APS-30G3 (c) and (B) LMP (a), APS-30G3 (b), APS-30G3-25LMP (c), and APS-30G3-50LMP (d).

To further investigate AW and pectin’s physicochemical interactions, the materials used in the fabrication of the composite films were analyzed by TGA. Figure 5A,B displays the derivative curves of all AW-based films plasticized with G3 and of the APS-based films plasticized with G3 with or without LMP, respectively.

Curves of thermal degradation included in Figure 5A showed two main degradation events. The first one, between 30 and 140 °C, is related to the evaporation of low-molecular-weight volatile compounds and physisorbed water molecules. The second step was found between 140 and 500 °C, with temperatures of maximum degradation rate, *T*_max_, at 294, 290, and 287 °C for AP-30G3, AS-30G3, and APS-30G3, respectively. This broad degradation step is associated with the overlapped thermal degradation of the polymers present in AW and of the plasticizer, G3 (*T*_max_ = 320 °C).^3^ Temperatures between 250 and 350 °C are usually associated in the literature with the thermal degradation of many organic extractives, such as waxes, alkaloids, carbohydrates, and so on. In particular, in this range occurs the thermal degradation of polysaccharides such as cellulose, hemicellulose, and starch, present in AW films.^52^ The small shoulder observed for AP-30G3 at *T* > 400 °C is related to the degradation of aromatic residues mainly present in lignin.^22,53^

The thermal behavior of the films significantly changed after the addition of LMP. Figure 5B shows the thermal degradation curves of APS-based films and their composites with 25 and 50 wt % LMP. LMP film thermal degradation curves were included for comparison. LMP presented two main degradation steps: the first one associated with the evaporation of water molecules (*T*_max_ = 45 °C) and the second one with the thermal decomposition of pectin chains (*T*_max_ = 222 °C). Temperatures of thermal degradation were similar to the ones previously reported by other authors.^51,54^

When LMP was incorporated into APS-30G3 films, the main peak associated with the thermal degradation of APS-30G3 at 287 °C splits into two peaks with *T*_max_ = 242 and 302 °C, probably indicating the presence of an LMP-rich face and an APS-rich face, respectively, similar to what happens when glycerol is incorporated as a plasticizer in thermoplastic starch^55^ or in hydrolyzed potato peel films.^3^ Then, with the increase in the amount of LMP to 50 wt %, the peaks got closer in temperature (249 and 275 °C), suggesting improved compatibility among components.

The addition of LMP to the AW-based films significantly improved the mechanical properties of the films (*p* > 0.05) (Table 3). The modulus and tensile strength of AP films increased by more than 800 and 400%, respectively, when 50 wt % LMP was added, also accompanied by an increase in the elongation at break. These results for AP-based films are in agreement with the results observed by SEM that showed a change from a porous cross section to a homogeneous and continuous one. Something similar was observed for AS and APS films, demonstrating one more time that pectin can be used as an excellent binding agent for biomass waste-derived bioplastics.^1^

The 50 wt % addition of LMP resulted in the best properties, and almost no significant differences were observed between AP, AS, and APS materials. Results were in agreement with what was observed by Otoni *et al.*, for films prepared with LMP and papaya puree,^56^ by Azaredo *et al.*, for pectin films with pomegranate juice, and by Martelli *et al.*, for banana puree films with incorporated pectin.^57^ According to them, the vegetable waste acts as a plasticizer in the highly brittle pectin materials whose individual mechanical properties were not possible to assay, decreasing the *E* and σ of their films. This effect was more noticeable for a higher proportion of vegetable waste in the composite.

Materials developed in this work with 50 wt % LMP showed mechanical properties comparable to the common polymers used in the food packaging industry. Their elastic modulus was superior to the ones of LDPE (*E* = 140–300 MPa), Mater-Bi (*E* = 161 MPa), and PCL (*E* = 200 MPa), and their tensile strength was similar to the ones of LDPE (σ = 7–17 MPa) and PLA (σ = 18 MPa) and superior to the one of Mater-Bi (σ = 8 MPa). Their elongation at break was superior to that of PS (ε_b_ = 2–3%) and PLA (ε_b_ = 9%).^41,58,59^

Results of WVP and WCA are also included in Table 3. Nonsignificant differences were found in the WVP of plasticized AW films and their composites with LMP, but it was observed that permeability values were higher for materials prepared with APs, compared to ASs or APSs. The absence of significant differences among materials with the addition of LMP could be related to the presence of an AP- or AS-rich face, which is responsible for the WVP values, according to the results of TGA.

Regarding their interaction with water droplets, a significant increase in the WCAs was observed after the addition of LMP. The WCAs were higher for the films based on APs or APSs than for the films based on ASs, in line with what was observed by XRD and UV–vis and in agreement with NMR polymer composition (AP has a superior content of fibers and aliphatic polyesters). Besides, LMP hydroxyl groups interact with polymers present in AW, as shown by FTIR and TGA, reducing the amount of available OH groups to interact with water molecules, increasing the surface hydrophobicity of the films.

## 4 Composite Films as Biodegradable and Active Food Packaging

**Figure 6:**
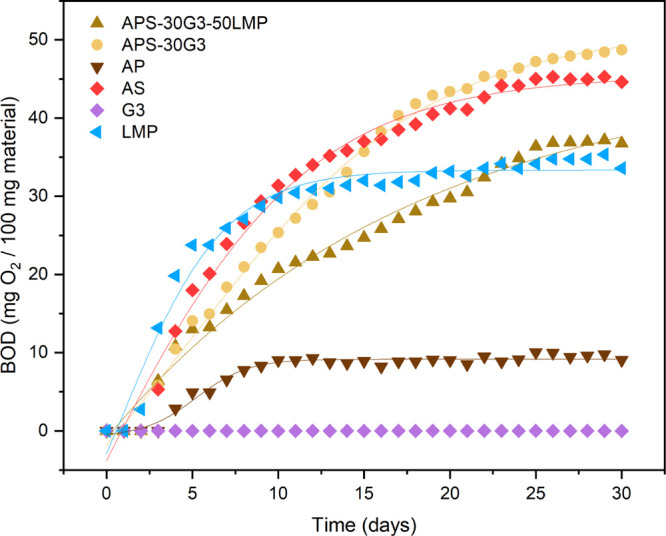
Biochemical oxygen consumption (mg O_2_/100 g material) as a function of time (days) for the APS-30G3-50LMP sample and its controls: APS-30G3, APs, ASs, G3, and LMP.

Since new sustainable food packaging materials are expected to be biodegradable like organic matter, the biochemical oxygen demand of AP powder, AS powder, LMP, APS-30G3 films, and APS-30G3-50LMP films was studied during a 30 day experiment, and the results are included in Figure 6 and Table S5. The films prepared with APSs were selected here because they represent a method of obtaining of bioplastics using the entire AW and thus contributing to the zero-waste and circular economy principles.

Table S5 shows the fitted curves’ parameters, being the meaningful parameter *A*2, which gives the extrapolated value for each curve’s plateau, indicating the maximum amount of oxygen consumed when all the biodegradable material is degraded (normalized on 100 mg of the material).

AP powder and LMP reached the plateau during the first week, while AS powder and APS-30G3 films reached the plateau at the end of the experiment. On the other hand, APS-30G3-50LMP films had slower biodegradation, and G3 alone did not undergo any biodegradation. This last result represents a controversial issue in the literature. While some authors claim that glycerol (G), the repeating unit of G3, is biodegradable, others consider it is not.^60−62^ The fact is that these compounds may be consumed by specific microbes present in the soil as carbon and energy sources under aerobic conditions^61^ or can be biodegraded *via* an anaerobic process.^63^ Results of G3 biodegradation in both soil and seawater are lacking, and more research should be carried out to clarify this issue.

The results observed for the BOD of bioplastics and AP and AS powders can be explained considering their different chemical compositions. For example, AS powder was much more biodegradable than AP powder, which can be associated with its superior starch component rather than cellulose.^64^ On the other hand, once hydrolyzed and plasticized, this difference disappeared. Indeed, APS-30G3 behaves similarly to ASs, suggesting that AP becomes much more biodegradable. Considering that both APs and ASs reduce their crystallinity during the process, it could be speculated that AP’s low degradation is due to cellulose’s crystallinity, while crystallinity of starch does not affect the biodegradability of ASs. Finally, APS-30G3-50LMP has a final value of around 46 mg O_2_/100 mg material, compared to the 51 mg O_2_/100 mg of APS-30G. However, this little difference may not be due to the physical-chemical effect of LMP. Indeed, from the test on LMP, 100 mg of LMP consumes 33 mg of O_2_ at the plateau, while 100 mg of APS-30G consumed 51 mg. In APS-30G3-50LMP, APS-30G3 and LMP are combined in a 1:1 proportion. Thus, the expected amount of O_2_ consumed at the plateau would be around 42 mg O_2_/100 mg material. Therefore, results suggest that the biodegradability of APS-30G3-50LMP is just the linear combination of the two components.

**Figure 7:**
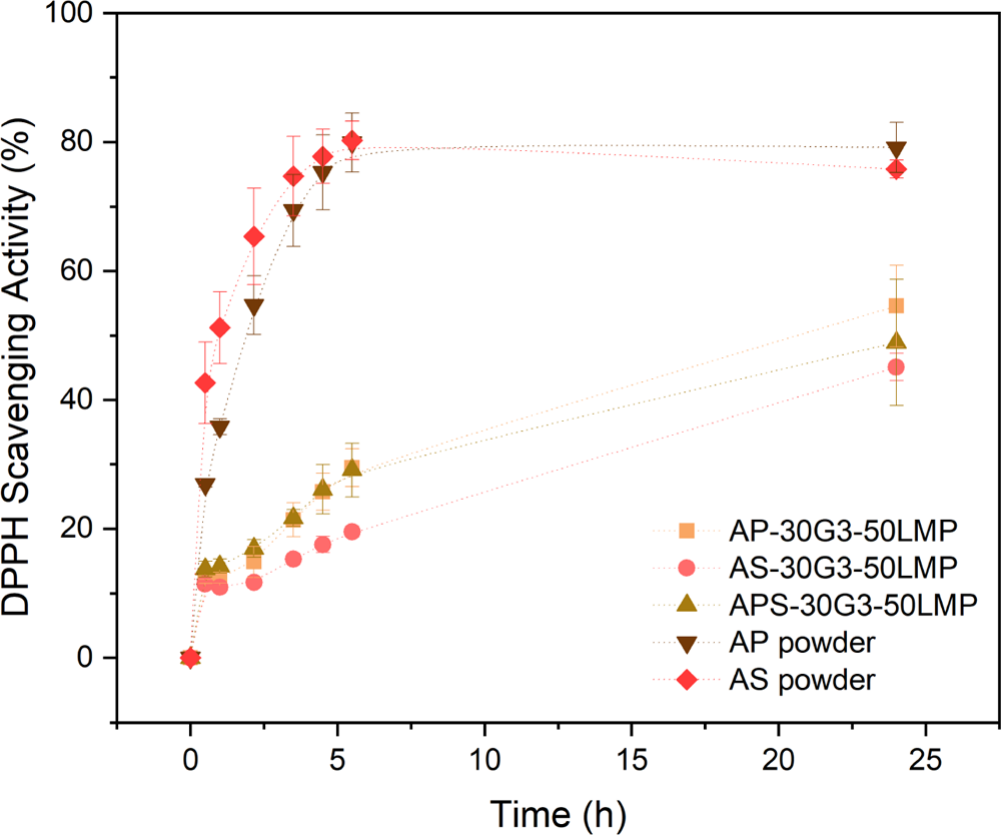
DPPH^•^ free radical scavenging activity (%) as a function of time (h) for AP and AS powders and their composites with 50 wt % LMP.

The DPPH^•^ is a stable free radical with an unpaired valence electron on a nitrogen atom. It is often used to carry out the standard test that serves to determine the antioxidant capacity of various materials since it changes from purple to yellow when it receives an electron or hydrogen radical, that is, when it is reduced.^47^ Both the APs and ASs have a wide variety of phytochemicals that function as antioxidants.^14^ In this work, it was found that the DPPH^•^ radical scavenging activity of the AP and AS powders did not present significant differences, as shown in Figure 7 and Table 3. Their antioxidant action increased over time, reaching the maximum at 5 h. Regarding the films with LMP, it was found that the release of antioxidant substances is prolonged in time and that the AS-30G3-50LMP showed the lowest scavenging activity at least until 24 h.

These antioxidant results are superior to the ones reported for gelatin films with papaya peel microparticles (0.84 μmol TE/g film)^65^ and starch films with the propolis extract (1.3 μmol TE/g film).^66^ Besides, the results reported here were comparable to the ones reported by Tran *et al.*(67) for poly(propylene carbonate) and cellulose acetate films loaded with oregano waste extract (4.1 μmol TE/g film) and by Quilez-Molina *et al.*(68) for PVA films with tea waste (4.5 μmol TE/g film), demonstrating a high antioxidant activity.

Due to their superior properties and in order to use both APs and ASs, materials prepared from APSs with 30 wt % G3 and 50 wt % LMP were selected for further analysis and evaluation of their suitability for food packaging in terms of O_2_P and migration of components in Tenax. Indeed, besides being biodegradable, a good packaging must protect food from oxidation. In this sense, the developed films presented antioxidant properties and excellent oxygen barrier properties, demonstrated by the low O_2_P determined in this work for APS-30G3-50LMP bioplastics: 22.5 ± 1.5 cm^3^ μm m^–2^ day^–1^ kPa^–1^. This value was similar to the values reported for starchy materials (O_2_P < 20 cm^3^ μm m^–2^ day^–1^ kPa^–1^) and even lower than the one reported for the high-density polyethylene (HDPE) material (42.7 cm^3^ μm m^–2^ day^–1^ kPa^–1^), traditionally used for food packaging.^69^

Finally, the overall migration analysis of APS-30G3-50LMP in Tenax gave a migration of 8.60 ± 0.45 mg/dm^2^, thus complying with the current legislation, which establishes a detection limit of 10 mg/dm^2.^^70^ This proves that blending the APS-30G3 with LMP under the mild conditions of the presented conversion process results in bioplastics that can be used to expand the field of applicability of the APSs for food packaging applications.

## 5 Conclusions

Avocado industrial processing byproducts represent an alternative exploitation source of bioactive compounds and starch. In this work, a sustainable method of obtaining bioplastics was developed and optimized using the AW: peels and seeds. The materials were obtained by combining the following processes: hydrolysis in a weak acid medium, plasticization, and mixing with the pectin polymer. The characterization of these materials revealed that their components were compatible and that they interacted by hydrogen bonds. In particular, the materials obtained by the combination of avocado peels and seeds plasticized by polyglycerol G3 and with an addition of 50 wt % pectin presented were proved to be the best candidates as food packaging films: suitable mechanical and barrier properties, antioxidant capacity, biodegradability, and adequately low migration of components in Tenax. Therefore, the developed materials represent a suitable and sustainable alternative to traditional nonbiodegradable plastic food packaging materials, standing out for their antioxidant activity, their natural composition, and their environmentally friendly method of development.

## Supporting Information Available

Materials’ chemical (NMR, XRD, and FTIR) and optical (UV–visible) properties, as well as information on material biodegradability (PDF)
