# Supplementary material for: Avocado Peels and Seeds: Processing Strategies for the Development of Highly Antioxidant Bioplastic Films
Source: ACS Appl Mater Interfaces. 2021 Aug 4;13(32):38688–99. doi: 10.1021/acsami.1c09433 (PMC8397233; doi:10.1021/acsami.1c09433)
Supplement: Supplementary file 1 — am1c09433_si_001.pdf [file am1c09433_si_001.pdf]

SUPPORTING INFORMATION

Avocado peels and seeds: Processing strategies for the development of highly antioxidant bioplastic films

Danila Merino[1,*], Laura Bertolacci[1], Uttam C. Paul[1], Roberto Simonutti[2], Athanassia Athanassiou[1,*]

[1]Smart Materials, Istituto Italiano di Tecnologia, Via Morego, 30, Genoa, 16163, Italy.

[2] Dipartimento di Scienza dei Materiali, Università di Milano-Bicocca, Via Roberto Cozzi 55, 20125, Milano, Italy

[*]*Corresponding author*: Dr. Danila Merino and Athanassia Athanassiou, Smart Materials Group, Italian Institute of Technology (IIT), Via Morego 30, 16163, Genoa, Italy. Tel: +39 010 28961. E-mail: danila.merino@iit.it; athanassia.athanassiou@iit.it

SI 1. Solid state NMR

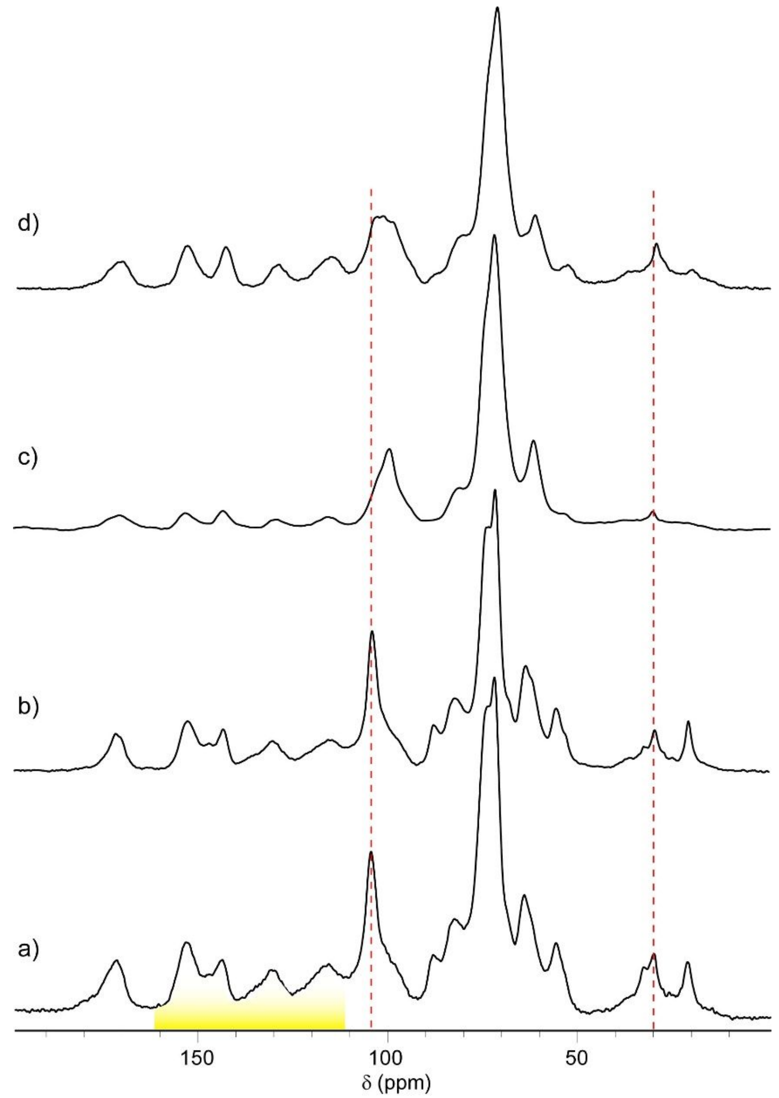

Figure S1 ^{13}C CPMAS spectra of (a) AP, (b) AP fiber, (c) AS; and (d) AS fiber. The resonances of lignin in AS are highlighted in yellow for sake of clarity. Vertical dotted red lines are guides for the eyes for the anomeric carbon of cellulose (at 105 ppm) and for methylenes of aliphatic polyesters (at 31 ppm).

In AP and AS systems, the signals due to carbohydrate polymers are the most intense peaks, in the spectral range from 110 to 105 ppm. Around 173 ppm a broad signal generated by carboxylic carbons, either as free acids or as esterified groups is evident. These carbons can be part of pectins or aliphatic polyesters. Aliphatic polyesters also generate signals around 30 ppm due to methylene carbons. The region between 115 and 160 ppm is specific of aromatic carbons and carbons on double bonds. Lignin is characterized by a quite complex tridimensional structure, but it is well accepted that the main building block are syringil (S) and guaiacyl (G) units [1]. The signal at 154 ppm is assigned to C-3 and C-5 of S units that are etherified at C-4. The signal at 144 ppm is also assigned to C-3 and C-5 of S units, but for those with free phenolic groups at C-4. Additionally, the signal at 144 ppm is assigned to C-3 and C-4 of G units. The signal at 130 ppm is assigned to C-1 and C-4 of S and G units that are etherified at C-4. The protonated carbons of syringyl (C-2 and C-6) units and guaiacyl units (C-2, C-5 and C-6) resonate around 116 ppm. Going more into the details of the carbohydrate signals, the chemical shift regions 104–107 ppm, 80–92 ppm and 60–67 ppm are attributed to the C1, C4, and C6 signals respectively of the glucose unit of the cellulose [2]. Close to the C1 signal of cellulose, at 101.8 ppm, C1 signal of hemicellulose is present. The other signals of hemicellulose are in part overlapped with cellulose signals and can be found as shoulders at lower chemical shift with respect to C6 signal of cellulose [3–5]. However also the C1 of galacturonic acid, component of the pectin, resonates around 98 ppm [6]. Finally, the C1 of starch can be found between 98 and 105 ppm depending on the degree of crystallinity and plasticization. Some of the authors have developed an approach for the determination of composition in biobased systems containing only cellulose, pectin, hemicellulose and aliphatic polyesters [7], this method exploits the intensity of signals univocally

assigned and necessary stochiometric relationships to make the calculations. The presence of starch does not allow the application of this approach; thus, samples devoid of starch have been prepared and analyzed (AP fiber and AS fiber). Comparing the spectra depicted in **Figure S1 (a-b)** it is clear that the avocado peel does not contain significative amount of starch, instead the spectrum of AS fiber is quite different from that of AS. Considering that the extraction protocol does not affect the relative of presence of the other component in AS fiber with respect to AS, the quantity of starch can be determined. For the quantification of lignin, the considerations discussed by Haw and coworkers [8] are taken into account, thus the sum of the intensities of all the signal due to the aromatic carbons (from 115 ppm to 160 pp) of lignin is compared with intensity of the anomeric carbons of the carbohydrate polymers.

SI 2. XRD

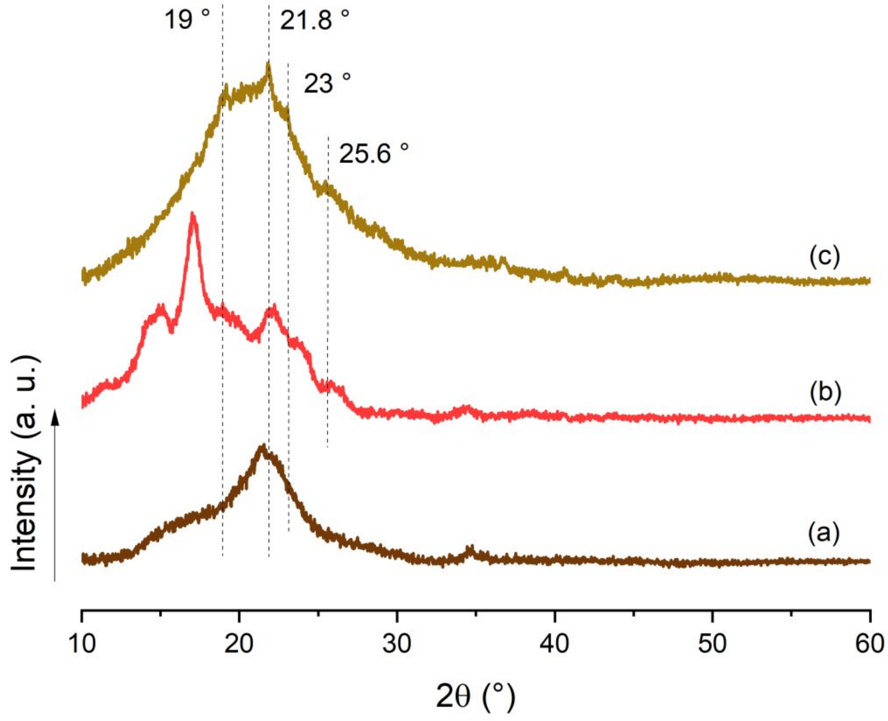

Figure S2 XRD patterns of AP powder (a), AS powder (b), and APS-30G3 film (c).

SI 3. FTIR

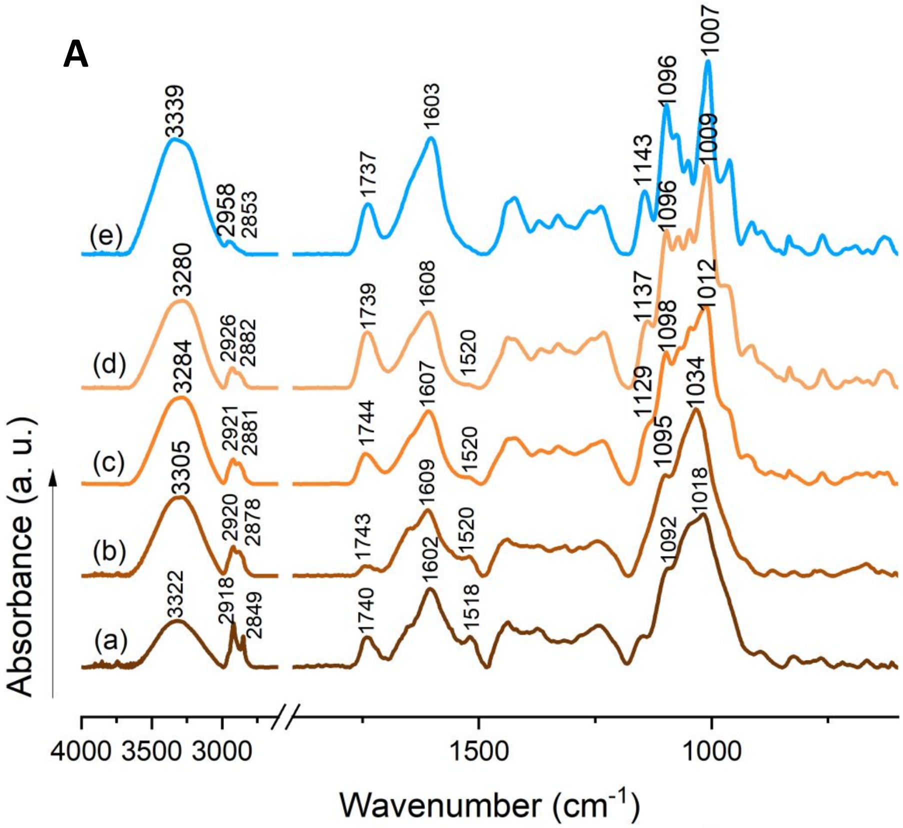
A
(e)
(d)
(c)
(b)
(a)
3339
2958
2853
1737
1603
1143
1096
1007
3280
2926
2882
1739
1608
1520
1137
1096
1009
3284
2921
2881
1744
1607
1520
1129
1098
1012
3305
2920
2878
1743
1609
1520
1095
1034
3322
2918
2849
1740
1602
1518
1092
1018
Absorbance (a. u.)
4000 3500 3000
1500
1000
Wavenumber (cm-1)

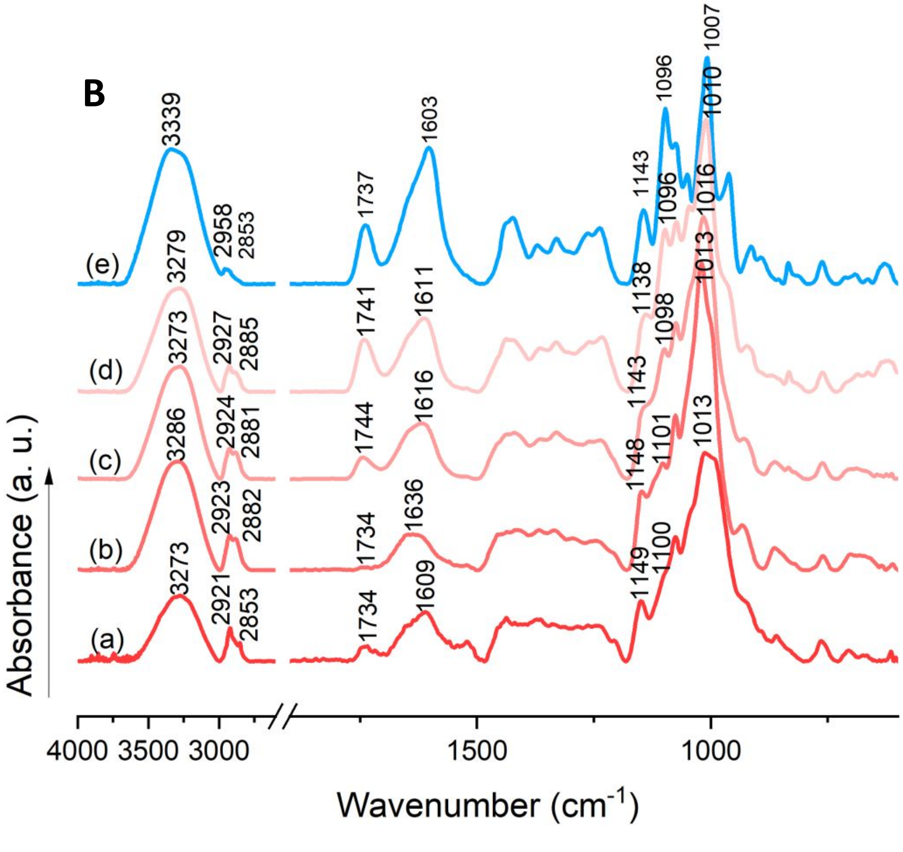
B
(e)
(d)
(c)
(b)
(a)
3339
2958
2853
1737
1603
1143
1096
1007
3279
2927
2885
1741
1611
1138
1096
1010
3273
2924
2881
1744
1616
1143
1098
1016
3286
2923
2882
1734
1636
1148
1101
1013
3273
2921
2853
1734
1609
1149
1100
1013
Absorbance (a. u.)
4000 3500 3000
1500
1000
Wavenumber (cm-1)

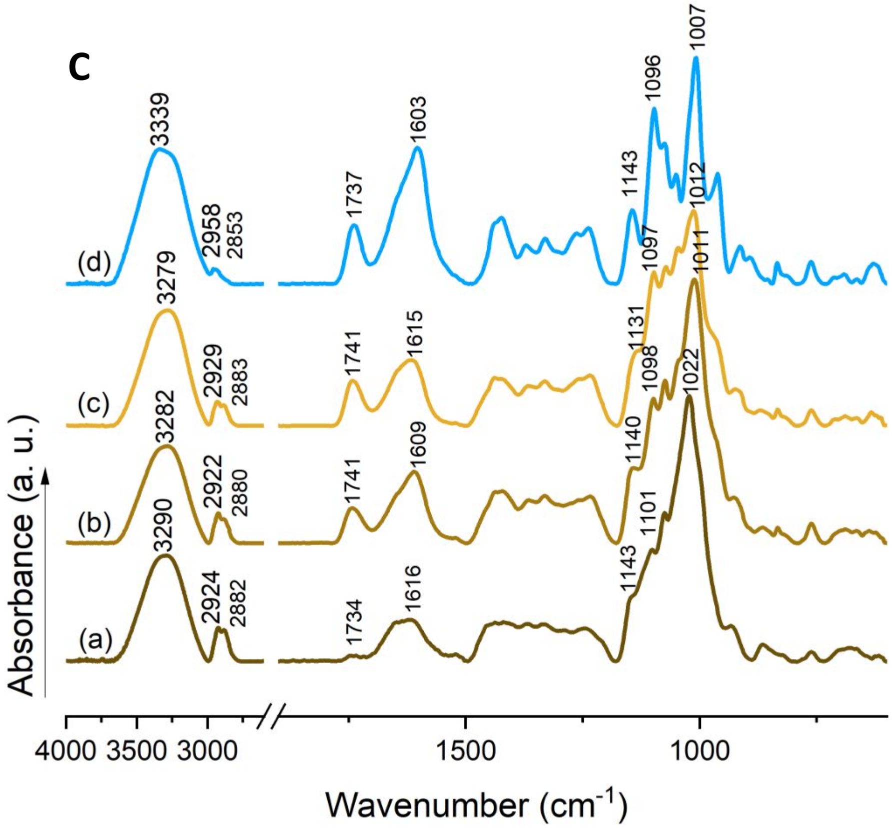

Figure S3 FTIR spectra of **A**: (a) AP powder, (b) AP-30G3, (c) AP-30G3-25LMP, (d) AP-30G3-50LMP and (e) Ca-LMP, **B**: (a) AS powder, (b) AS-30G3, (c) AS-30G3-25LMP, (d) AS-30G3-50LMP and (e) Ca-LMP, and **C**: (a) APS-30G3, (b) APS-30G3-25LMP, (c) APS-30G3-50LMP and (d) Ca-LMP.

SI 4. Optical properties

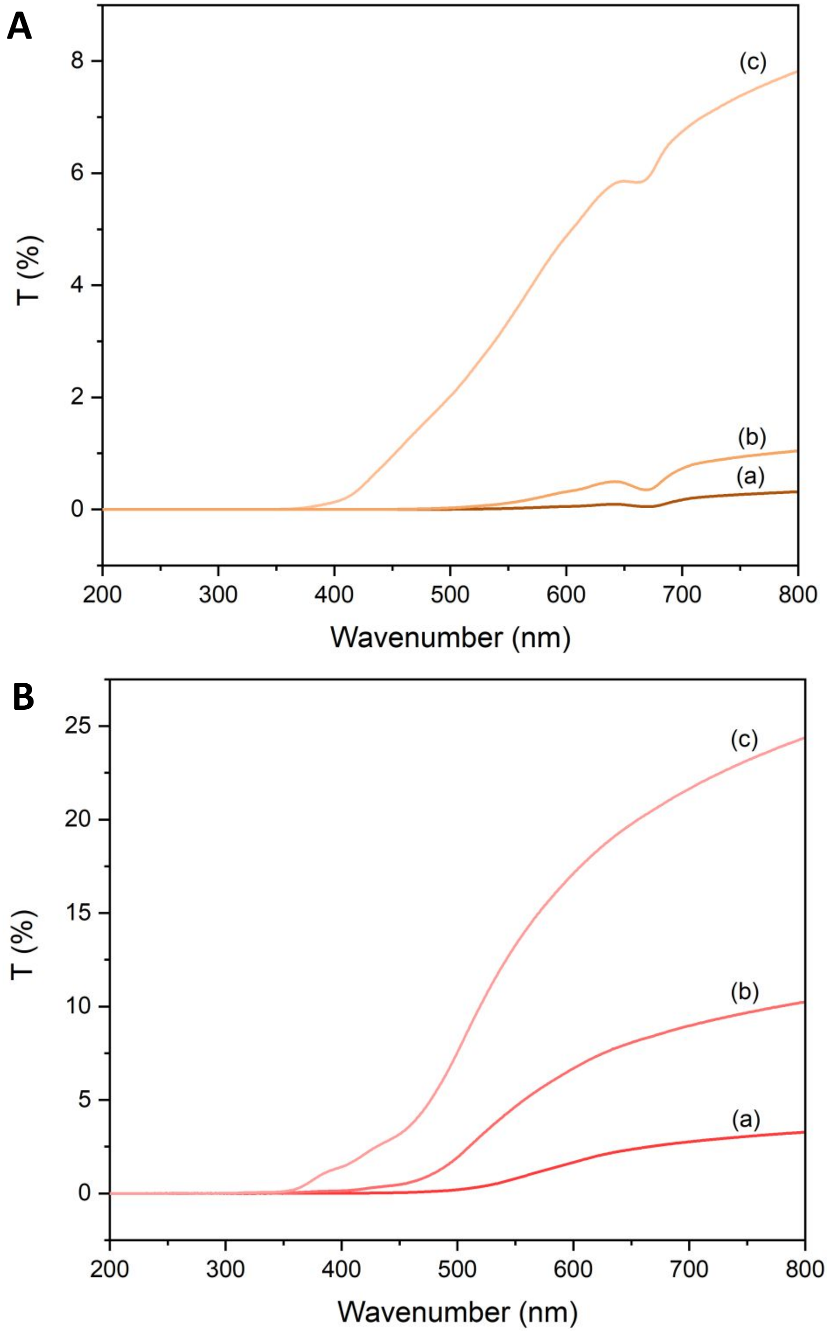

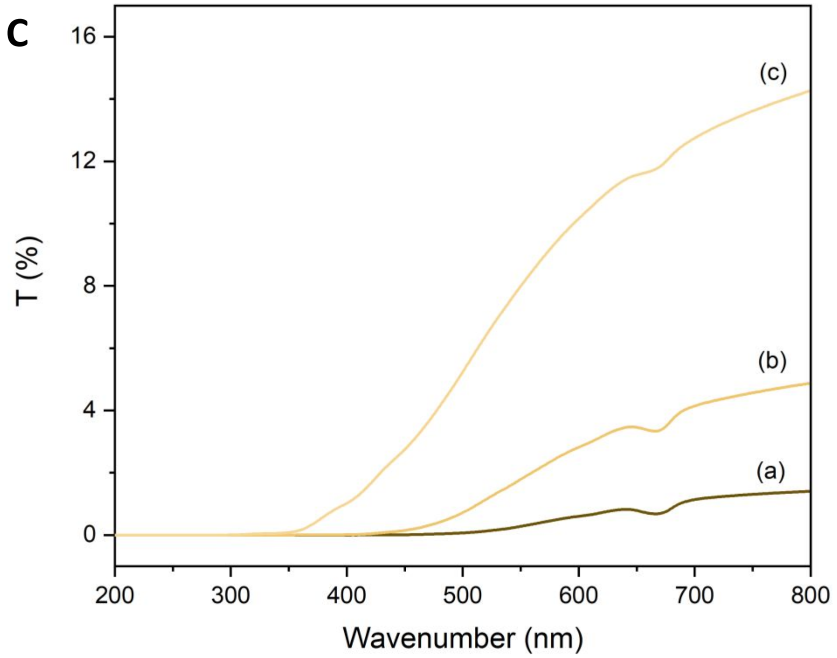

Figure S4. Transmittance spectra of **A**: (a) AP-30G3, (b) AP-30G3-25LMP and (c) AP-30G3-50LMP , **B**: (a) AS-30G3, (b) AS-30G3-25LMP and (c) AS-30G3-50LMP, and **C**: (a) APS-30G3, (b) APS-30G3-25LMP and (c) APS-30G3-50LMP

SI 5. BOD table

Table S5 Fitting of curves of BOD vs. Time for APS-30G3-50LMP, APS-30G3, AP, AS, G3 and LMP. A2 parameter gives the BOD value at which the plateau is reached.

**Model**	**DoseResp**					
**Equation**	y = A1 + (A2-A1)/(1 + 10^((LOGx0-x)*p))					
**Plot**	APS-30G3-50LMP	APS-30G3	AP	AS	G3	LMP
**A1**	-15875.20185 ± 4084846.89229	-29.60773 ± 9.20315	-0.75578 ± 0.53168	-64.37209 ± 41.74751	0 ± --	-30.03637 ± 20.21159
**A2**	**46.18505 ± 8.49327**	**51.32776 ± 1.103**	**9.1465 ± 0.12173**	**45.4595 ± 1.01093**	**0 ± --**	**33.32999 ± 0.45803**
**LOGx0**	-102.48402 ± 4608.2652	4.60885 ± 1.55606	5.1951 ± 0.28381	-1.28832 ± 4.20837	6 ± --	0.86851 ± 1.89326
**p**	0.02466 ± 0.01941	0.06111 ± 0.00646	0.28608 ± 0.04155	0.06967 ± 0.01147	-0.16667 ± --	0.14451 ± 0.02558
**Reduced Chi-Sqr**	2.10845	1.31107	0.2846	2.89546	0	2.99217
**R-Square (COD)**	0.98581	0.99556	0.976	0.98785	--	0.97357
**Adj. R-Square**	0.98424	0.99507	0.97333	0.9865	0.9865	0.97063
